# Degradation of Phosphorylated p53 by Viral Protein-ECS E3 Ligase Complex

**DOI:** 10.1371/journal.ppat.1000530

**Published:** 2009-07-31

**Authors:** Yoshitaka Sato, Takumi Kamura, Noriko Shirata, Takayuki Murata, Ayumi Kudoh, Satoko Iwahori, Sanae Nakayama, Hiroki Isomura, Yukihiro Nishiyama, Tatsuya Tsurumi

**Affiliations:** 1 Division of Virology, Aichi Cancer Center Research Institute, Chikusa-ku, Nagoya, Japan; 2 Department of Virology, Nagoya University Graduate School of Medicine, Nagoya, Japan; 3 Division of Biological Science, Graduate School of Science, Nagoya University, Nagoya, Japan; University of Wisconsin-Madison, United States of America

## Abstract

p53-signaling is modulated by viruses to establish a host cellular environment advantageous for their propagation. The Epstein-Barr virus (EBV) lytic program induces phosphorylation of p53, which prevents interaction with MDM2. Here, we show that induction of EBV lytic program leads to degradation of p53 via an ubiquitin-proteasome pathway independent of MDM2. The BZLF1 protein directly functions as an adaptor component of the ECS (Elongin B/C-Cul2/5-SOCS-box protein) ubiquitin ligase complex targeting p53 for degradation. Intringuingly, C-terminal phosphorylation of p53 resulting from activated DNA damage response by viral lytic replication enhances its binding to BZLF1 protein. Purified BZLF1 protein-associated ECS could be shown to catalyze ubiquitination of phospho-mimetic p53 more efficiently than the wild-type in vitro. The compensation of p53 at middle and late stages of the lytic infection inhibits viral DNA replication and production during lytic infection, suggesting that the degradation of p53 is required for efficient viral propagation. Taken together, these findings demonstrate a role for the BZLF1 protein-associated ECS ligase complex in regulation of p53 phosphorylated by activated DNA damage signaling during viral lytic infection.

## Author Summary

Inhibition of p53-mediated transactivation is essential for regulating the cellular environment advantageous for viral infection. Specially, DNA viruses target p53 for inactivation through the ubiquitin-proteasome pathway. The E6 protein of the high-risk human papillomaviruses and the cellular ubiquitin-protein ligase E6AP form a complex which causes ubiquitination and degradation of p53. The adenovirus E1B 55-kDa protein binds to both p53 and E4orf6, and recruits a Cullin-containing complex to direct the ubiquitin-mediated proteolysis of p53. However, in comparison with the effects of the smaller DNA viruses, much less is known regarding the precise mechanisms whereby the Epstein-Barr virus (EBV) inhibits functions of p53. EBV possesses two alternative life cycles, latent and lytic replication. In latent phase, p53 is regulated by MDM2 ubiquitin ligase while after induction of lytic replication p53 is phosphorylated and the level of activated p53 is regulated by a novel system independent of MDM2. This report describes a unique functional role of the BZLF1 protein encoded by EBV in the modulation of activated p53. In this pathway, BZLF1 protein serves as an adaptor molecule for both Cul2- and Cul5-containing E3 ubiquitin ligase complexes to stimulate the ubiquitination and degradation of p53 for inhibiting apoptosis, indicating redundancy in the EBV machinery to downregulate p53 level. Therefore, it would be possible that the complexes regulate not only p53 but also various proteins that interact with BZLF1 protein.

## Introduction

The tumor suppressor p53 plays an important role in maintaining genomic integrity [1],[2]. In unstressed normal cells, p53 usually exists in a hypophosphorylated form at only low levels due to rapid degradation through the ubiquitin-dependent proteasome pathway [3]. MDM2 is a key regulator of turnover by binding to p53 and promoting its ubiquitination by acting as an E3 ubiquitin ligase. In response to DNA damage, p53 is phosphorylated at S15 by ataxia-telangiectasia mutated (ATM) and then T18 by casein kinase 1, preventing the interaction with MDM2, subsequently leading to escape from proteasomal degradation [4]. The p53 protein level becomes elevated, resulting in an increase in p53-dependent transcription of its target genes, subsequently leading to cell cycle arrest or apoptosis [5],[6].

Ubiquitination is important for the regulation of a variety of cellular processes, including signal transduction, development, apoptosis, cell cycle progression, and the immune response [7],[8],[9]. The ubiquitination of a substrate requires a cascade of enzymatic reactions involving an E1 activating enzyme, an E2 conjugating enzyme, and finally an E3 ligase enzyme that covalently attaches ubiquitin to a lysine residue of the target protein [10]. The latter enzyme is the most diverse, demonstrating substrate specificity and determining the rate of ubiquitin conjugation. The E3 ligase itself can be either a single protein or a multiprotein complex. Cullin-containing ligases constitute a large class of E3s [11], primarily consisting of a substrate-specific adaptor protein, the scaffold protein Cullin, and a RING finger-containing protein that interacts with E2 ligase [12],[13],[14].

For evading host security responses and generating an advantageous environment for viral replication, a number of viruses have evolved sophisticated mechanisms to utilize or manipulate the host ubiquitin system. Specially, DNA viruses target p53 for inactivation through the ubiquitin-proteasome pathway. The E6 protein of the high-risk human papillomaviruses and the cellular ubiquitin-protein ligase E6AP form a complex which causes ubiquitination and degradation of p53 [15]. The adenovirus E1B 55-kDa protein binds to both p53 and E4orf6, and recruits a Cullin-containing complex to direct the ubiquitin-mediated proteolysis of p53 [16]. However, in comparison with the effects of the smaller DNA viruses, much less is known regarding the precise mechanisms whereby the Epstein-Barr virus (EBV) inhibits transcriptional functions of p53.

EBV, a human gamma-herpesvirus, is associated with several B-cell and epithelial-cell malignancies and can choose between two alternative infection states; latent and lytic [17]. Infection is primarily latent, but EBV periodically reactivates and replicates in a lytic manner in a subset of B cells, which is essential for viral propagation and transmission. The switch from latent to lytic infection is triggered by the BZLF1 protein [18], a b-Zip transcriptional factor which binds to the promoters of early lytic genes [19],[20]. Induction of the EBV lytic program elicits a cellular DNA damage response with activation of the ATM-dependent DNA damage signal transduction pathway [21]. Although ATM-dependent DNA damage signaling is activated and consequently p53 is phosphorylated at various sites including S15, the levels of p53-downstream targets are maintained at very low levels especially at middle to late stages of the infection [21],[22]. This poses the fascinating puzzle of why p53-downstream signaling is blocked in EBV lytic infection even when p53 is phosphorylated through activation of the ATM-mediated DNA damage response. Recently, we found that the BZLF1 protein induces p53 degradation during the lytic infection [23]. In this study, we investigated the mechanisms by which the viral immediate-early (IE) BZLF1 protein targets p53 for degradation and revealed that the protein directly functions as an adaptor component of the ECS (Elongin B/C-Cul2/5-SOCS-box protein) ubiquitin ligase complex targeting phosphorylated p53 for degradation during the lytic replication.

## Results

### The EBV BZLF1 protein constitutes the ECS ubiquitin ligase complex through interaction with Cul2/Cul5

**Figure 1:**
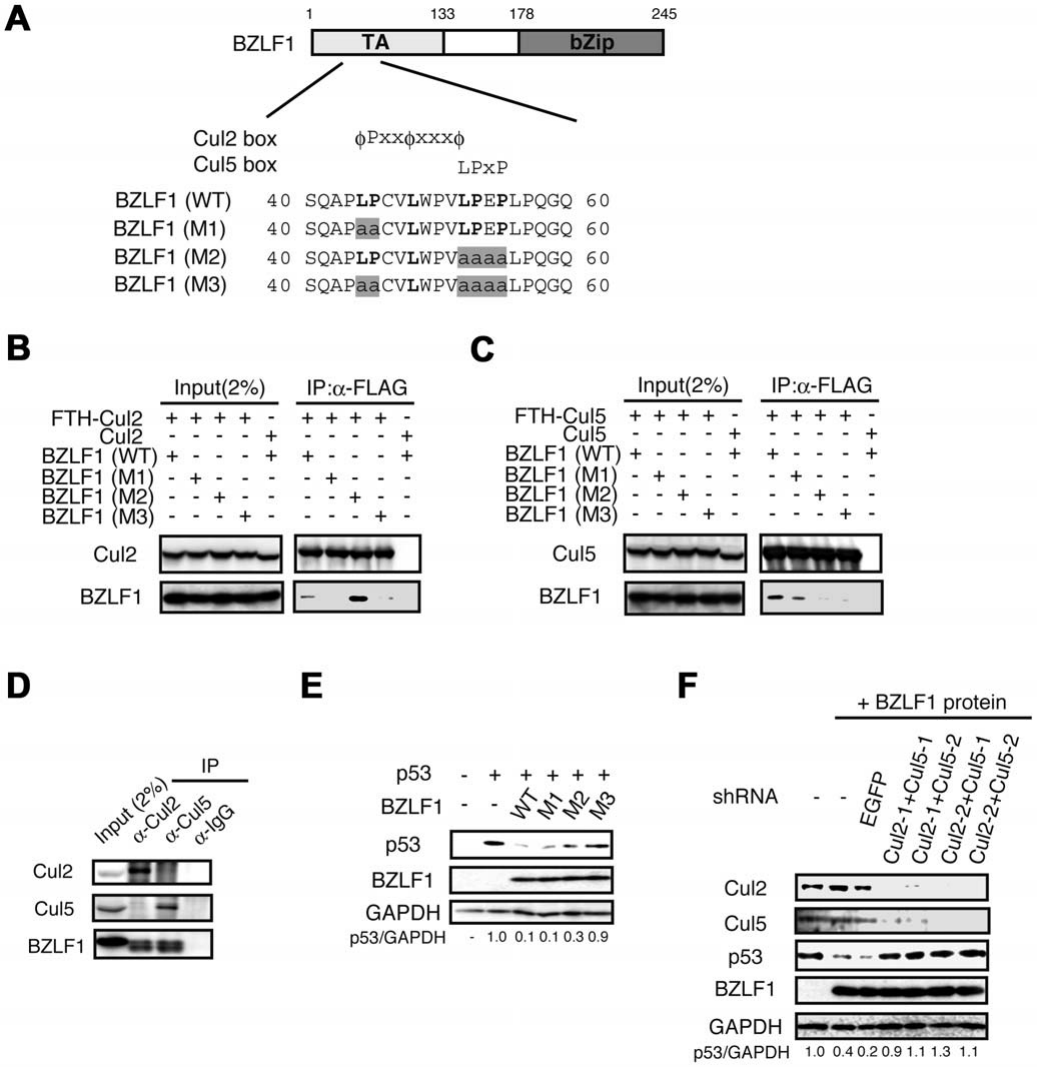
BZLF1 protein associates with Cul2 and Cul5 through the Cul-box motif. (A) BZLF1 protein contains putative Cul2-box and Cul5-box motifs at the N-terminus. Conserved amino acids are indicated in bold. TA, transactivation domain; bZip, basic leucine-zipper domain. Mutated amino acids are highlighted. (B and C) Lysates of 293T cells transiently expressing the indicated proteins were subjected to immunoprecipitation followed by immunoblotting with the indicated antibodies. (D) BZLF1 protein interacts with both Cul2 and Cul5 in lytic replication-induced cells. The lytic infection was induced by transfection with BZLF1 protein expression plasmid into 293/EBV cells. Cells were treated with MG132 for 4 h before harvesting and then harvested 48 h post-transfection. IP and IB assays were performed using the indicated antibodies. (E) The BZLF1 M3 mutant features relaxed reduction of p53. SaOS-2 cells were transfected with BZLF1 wild-type or its mutant expression plasmid, and p53 expression plasmids as indicated. Lysates were applied for immunoblot analysis with each antibody indicated. The intensity of the bands is expressed as a ratio between p53 and GAPDH. (F) Cul2 and Cul5 are required for p53 degradation. 293/EBV cells were co-transfected with plasmids expressing BZLF1 protein and shRNA targeting Cul2 and Cul5 mRNAs. Cells were lysed 48 h post-transfection for IB analysis with the indicated antibodies.

We performed a bioinformatics search for structural motifs within the BZLF1 protein that interact with the established ubiquitin E3 ligase, to identify the E3 ligase responsible for the BZLF1 protein-mediated degradation of p53 during lytic infection [23]. Since the BZLF1 protein does not possess a RING finger domain, it might not have an intrinsic E3 ligase activity by itself. By this approach, we found that the BZLF1 protein possesses putative Cul2 and Cul5 binding motifs termed the Cul2-box and Cul5-box [14],[24], respectively (Figure 1A). Cul2 and Cul5 are subunits of the well-defined ECS ubiquitin ligase complex [14]. To investigate whether the BZLF1 protein can associate with Cul2 and Cul5, three BZLF1 protein mutants (M1-M3) were constructed (Figure 1A): M1, which contains mutations at positions 44 and 45 in the Cul2 box (LP to AA); M2, which contains mutations at positions in the Cul5 box (LPEP to AAAA); M3, which contains mutations in both Cul2- and Cul5- boxes. As shown in Figure 1B and S1A, a series of IP assays using tagged protein showed wild-type BZLF1 protein to interact with both Cul2 and Cul5. In contrast, the M1 mutant did not interact with Cul2 while the M2 mutant lacked any reaction with Cul5. These observations strongly suggest that the Cul2/Cul5-boxes in the N-terminus of BZLF1 protein are important for the physical association between the BZLF1 protein and Cul2/Cul5 E3 ligases.

To specifically address the question of whether BZLF1 protein interacts with Cul2 and Cul5 during lytic infection, IP assays were performed. The BZLF1 protein actually associated with both Cul2 and Cul5 and formed to ECS complex in lytic replication-induced 293/EBV cells (Figure 1C and S1B). These interactions were also confirmed in lytic infection-induced B95-8 cells (Figure S1C).

Next, we examined if the BZLF1 M1-M3 mutants could induce reduction of p53 level. Contrary to wild-type BZLF1 and M1, M2 mutants, the M3 mutant failed to decrease the level of p53 (Figure 1D).

To further investigate whether the BZLF1 protein-mediated degradation of p53 is dependent on the ECS complex, we applied RNAi experiments. Two independent sequences for each molecule were used as targets of RNAi. Since gene sequences for marmoset Cul2 and Cul5 could not be obtained from the NCBI database, we used 293/EBV cells to examine the effects of RNAi-knockdown of Cul2 and Cul5. As shown in Figure 1E, transfection of various combinations of plasmids encoding shRNAs specific for Cul2 and Cul5 mRNAs, as well as a BZLF1 expression plasmid for induction of lytic infection, caused significant increase in the level of p53, compared with that apparent in cells transfected with either a control plasmid for EGFP shRNA or an empty plasmid. Consistent with the findings in Figure 1D, we indeed confirmed that a knockdown of either Cul2 or Cul5 did not alter the p53 abundance in the lytic infection (data not shown). Hence, these results indicate that BZLF1 protein-mediated degradation of p53 is dependent on the ECS complexes.

### p53 ubiquitination mediated by recombinant BZLF1 protein-associated ECS complexes

**Figure 2:**
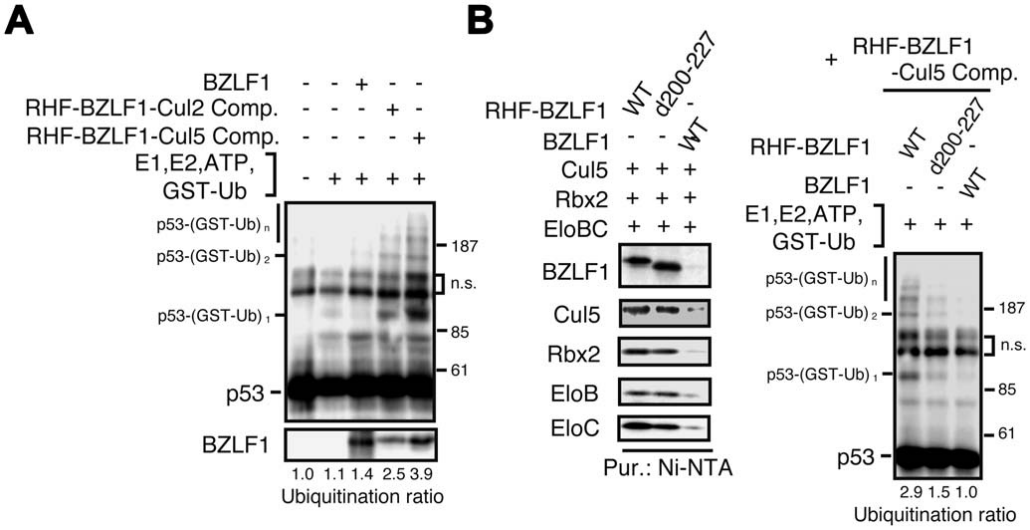
Ubiquitination of p53 by recombinant BZLF1-ECS complexes in vitro. (A) Recombinant BZLF1 protein, and BZLF1-EC_2_S or BZLF1-EC_5_S complexes were assayed for their ability to mediate the ubiquitination of p53 in the presence of ATP, Uba1 (E1), UbcH5c (E2) and GST-Ub. Reaction mixtures were incubated for 1 h at 26°C, boiled in SDS sample buffer, and then subjected to IB analysis with anti-p53 antibody. Intensity of the polyubiquitin chains is expressed as a ratio between polyubiquitinated p53 protein and p53 protein input. (B) The BZLF1 protein acts as an adaptor protein to recognize the substrate for p53 ubiquitination. Wild type BZLF1 (WT) or d200-227 mutant protein was expressed with components of the EC_5_S complex in Sf21 cells, and cell lysates were subjected to Ni-NTA affinity purification. The purified BZLF1 complexes were applied to the reaction and then IB analysis with anti-p53 antibody (right panels). Each recombinant BZLF1-EC_5_S complex purified from Sf21 insect cell lysates was analyzed by IB with the indicated antibodies (left panels).

To demonstrate that the BZLF1-ECS complex directly promotes p53 ubiquitination in vitro, we reconstituted complexes in Sf21 cells and assayed purified preparations for stimulation of p53 ubiquitination. Sf21 insect cells were coinfected with a series of recombinant baculoviruses encoding each subunit of the BZLF1-Cul2 complex (N-terminal His-and Flag-tagged BZLF1 protein (RHF-BZLF1), myc-Rbx1, HA-Cul2, Elongin B and Elongin C), or the BZLF1-Cul5 complex (RHF-BZLF1, 3×myc-Rbx2, HA-Cul5, Elongin B and Elongin C), and the resultant BZLF1-ECS complexes were purified [25]. Incubation of purified p53 substrate with RHF-BZLF1-Cul2 or RHF-BZLF1-Cul5 complexes resulted in ubiquitination of p53 in the presence of E1, E2, GST-ubiquitin and ATP, whereas purified BZLF1 protein alone did not process activity for ligating ubiquitin (Figure 2A). Omitting BZLF1 protein from the ECS complex abolished p53 ubiquitination and the RHF-BZLF1-Cul2/Cul5 complex catalyzed the ubiquitination of p53 in a dose-dependent manner (Figure S2A). To further confirm p53 ubiquitination by the BZLF1 protein-associated ECS complex, we performed time-course and drop-out experiments. Ubiquitinated p53 increased in a time-dependent manner (Figure S2B and C) and was not detected without E1, E2 or ATP (Figure S2D and E). Substitution of the d200-227 mutant BZLF1 protein, lacking the interaction with p53, for the wild-type in the RHF-BZLF1-Cul2/Cul5 complex eliminated the enhanced p53 ubiquitination (Figure 2B and S2F). Thus, the findings suggest that the BZLF1 protein recruits p53 to the ECS ligase complex for polyubiquitination, functioning as an adaptor for substrate recognition in the complex.

### Phosphorylation-dependent regulation of physical interaction and ubiquitination of p53 with the BZLF1 protein

**Figure 3:**
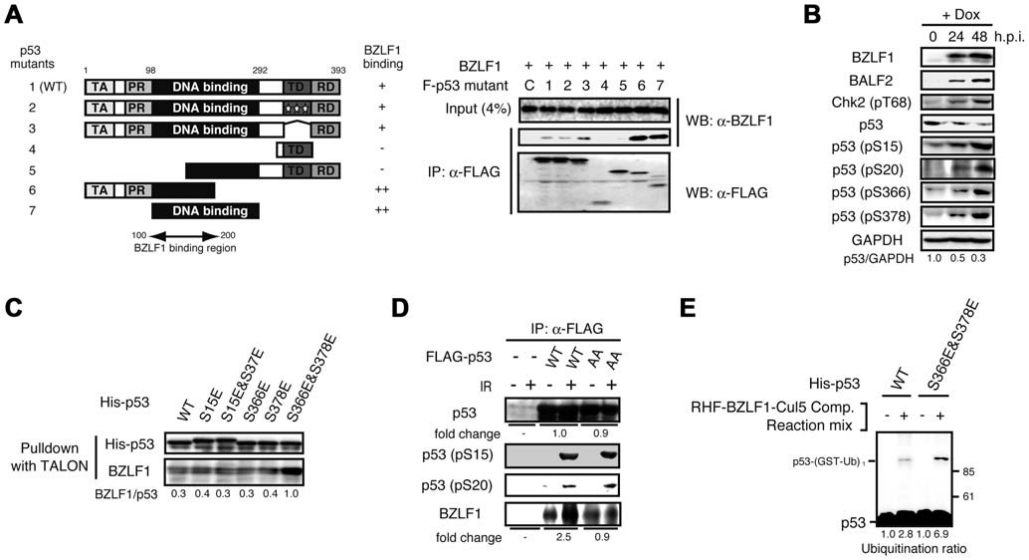
Phosphorylation-dependent enhancement of the interaction between p53 and BZLF1 protein. (A) BZLF1 protein interacts with the DNA-binding domain near the N-terminal of p53. SaOS-2 cells were cotransfected with BZLF1 protein and a series of FLAG-tagged p53 deletion mutant expression plasmids as indicated. Cell lysates were prepared 24 h post-transfection, and equal amounts of proteins under each condition were incubated with anti-FLAG M2 antibody beads. Immunoprecipitated proteins were analyzed by immunoblotting with anti-BZLF1 protein antibodies. Schematic illustration of the p53 wild-type and deletion mutants used. TA, transactivation domain; PR, proline rich domain; TD, tetramerization domain; RD, regulatory domain. (B) p53 is hyperphosphorylated during lytic infection. Tet-BZLF1/B95-8 cells were cultured with Dox for the indicated times. For p53 phosphorylation at S366 and S378 residues, IP/IB analysis was performed, while for the others IB analysis with the indicated specific antibodies was carried out. The p53/GAPDH ratio is provided. (C) Interaction of the BZLF1 protein with WT p53 and a variety of phospho-mimetic mutants. His-tagged p53 proteins were first attached to TALON beads and then incubated with purified recombinant BZLF1 protein in PBS. The beads were washed and spun down. Bound BZLF1 protein was detected by IB with anti-BZLF1 antibody. The intensity of the bands is expressed as a ratio between BZLF1 protein and p53. (D) The requirement of the p53 phosphorylation at C-terminus for the enhancement of binding to BZLF1 protein. 293T cells were transfected with FLAG-p53 (WT or S366A&S378A) expression vector, exposed to ionizing radiation (20 Gy) 46 h post-transfection and harvested 48 h post-transfection. FLAG-tagged p53 proteins were first purified using anti-FLAG antibody resin from cell extracts, and then incubated with purified recombinant BZLF1 protein. The beads were washed and spun down. Bound protein was detected by IB with indicated antibodies. AA indicates p53 S366A&S378A mutant. The band intensity was quantified as a fold change of IR-treated/untreated. (E) The phospho-mimic p53 mutant is more ubiquitinated than wild-type p53 by BZLF1- EC_5_S complex in vitro. Recombinant His-p53 protein (WT or S366E&S378E) was incubated in a reaction mixture containing purified BZLF1-EC_5_S complex. Reactions for p53 ubiquitination were carried out as described in the legend for Figure 2. The ubiquitinated p53/p53 ratio is indicated.

The mechanisms underlying regulation of the association between p53 and BZLF1 protein in cells is of considerable interest. Although the association between full-length p53 and BZLF1 protein is well-characterized [21],[23],[26], the domain of p53 interacting with the BZLF1 protein remains obscure. To address this, the interaction domain of p53 with the BZLF1 protein was found to be located within the DNA binding domain from IP analyses with deletion mutants of p53 (Figure 3A). Furthermore, the BZLF1 protein appeared to have affinity for C-terminus truncated mutants rather than full-length p53.

Interactions between certain classes of ubiquitin-ligating enzymes and their targets are tightly regulated by posttranslational modifications such as phosphorylation [27]. The ATM-Chk2 DNA damage signaling pathway is activated in the EBV lytic phase [21] and it was recently reported that Chk2-mediates phosphorylation of p53 at S366 and S378 in response to genotoxic stress [28]. These prompted us to assume that Chk2 might play a pivotal role in phosphorylation of p53 during lytic infection. Indeed, p53 was found to be phosphorylated at least at S15, S20, S366 and S378 with progression of EBV lytic infection (Figure 3B). However, the presence of significant redundancy should be kept in mind since the same p53 residue can be phosphorylated by several different kinases [29].

Based on structural analysis of p53 [30], it is speculated that the DNA binding domain is masked by its C-terminal regulatory domain rich in basic amino acids. Furthermore, phosphorylation of the C-terminal regulatory domain results in increased binding to DNA [31]. Thus, it is reasonable to assume that conformational change induced by phosphorylation of the regulatory domain enhances the association with the BZLF1 protein. To check this hypothesis, phospho-mimetic mutants of p53 (S15E, S15E&S37E, S366E, S378E, and S366E&S378E) His-tagged at the N-terminus were expressed in *E. coli* and purified. In vitro pull-down assays with His-p53 wild-type or mutants as bait were then performed using TALON His-tag affinity resin. As shown in Figure 3C, the bacterially expressed p53 S15E, S15E&S37E, S366E and S378E mutants showed negligible differences from wild-type p53 in their affinity for the BZLF1 protein. In sharp contrast, double mutations of S366 and S378 residues stimulated binding to the BZLF1 protein. To confirm the role of these p53 C-terminal phosphorylations, we generated p53 mutant in which residues 366 and 378 to nonphosphorylatable alanine. The p53 protein (WT or S366A&S378A) was expressed in 293T cells, phosphorylated by ionizing irradiation (IR), purified by anti-FLAG antibody and pull-downed with recombinant BZLF1 protein. In contrast to wild-type, p53 S366A&S378A mutant abolished the enhanced binding to BZLF1 protein under the IR stress (Figure 3D). These results imply that the phosphorylation of p53 at both S366 and S378 stimulates the association between p53 and BZLF1 protein.

To further test whether phosphorylation-mediated enhancement of the association affects p53 ubiquitination, we performed in vitro ubiquitination assays. As shown in Figure 3E and S2G, BZLF1 protein-associated ECS complexes more efficiently ubiquitinated S366E&S378E mutant as compared to the wild-type p53. Taken our results together, the C-terminal phosphorylation of p53 by Chk2 appears to stimulate ubiquitination through increase in the binding affinity for the BZLF1 protein.

### The degradation of p53 is required for efficient viral propagation

**Figure 4:**
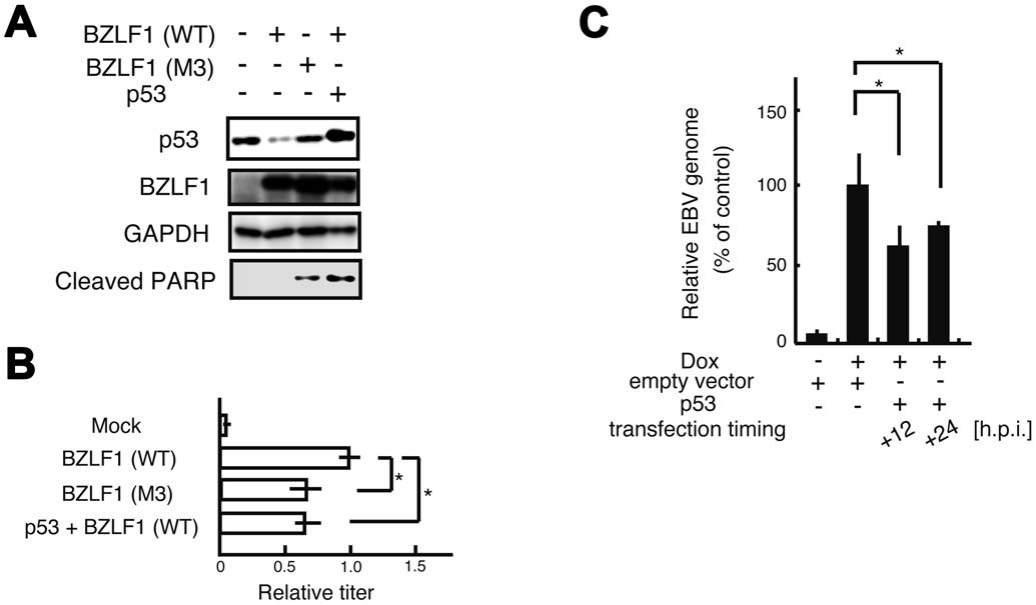
The degradation of p53 during lytic infection is required for efficient viral propagation. (A) Inhibition of p53 degradation in lytic replication induces apoptosis. The lytic infection was induced by transfection with BZLF1 protein expression plasmid (WT or M3) into 293/EBV cells. Cells were lysed 48 h post-transfection for IB analysis with the indicated antibodies. (B) The expression of p53 reduced the virus yield. The 293/EBV cells were transiently transfected with expression vectors as indicated. The virus yields were determined by counting GFP positive Akata (-) cells. The results are the average of three independent experiments and shown as values relative to the virus yield of BZLF1 (WT) (infectivity value of 1). Asterisk indicates *p*<0.05. (C) Ectopic expression of p53 at the middle and late stages of lytic infection interferes with efficient viral DNA replication. Tet-BZLF1/B95-8 cells were transfected with p53 expression plasmid using a Microporator at different timings as indicated and then cultured in the presence of doxycycline for 48 h. Viral DNA synthesis was determined by slot blot assay. Asterisk indicates *p*<0.05.

Induction of lytic replication by wild type BZLF1 protein results in low level of p53, while induction by the M3 mutant did not reduce the level (Figure 4A). It turned out that the inhibition of p53 degradation by the M3 mutant increased PARP cleavage, well-defined apoptotic marker (Figure 4A). We further analyzed the virus yield from the lytic-induced 293/EBV cells transfected by BZLF1 wild type or M3 mutant expression vector. As shown in Figure 4B, the yield of infectious virus from the BZLF1 M3 mutant expressed cells was poor than that of virus from wild-type BZLF1 expressed cells. The simultaneous transfection of both p53 and BZLF1 protein also produced low yield of infectious virus. To further assess whether the degradation of p53 during lytic infection is linked to anticipate effects on virus production, we examined temporal linkage of the p53 effect on viral DNA replication. The compensation for p53 at the middle and late stages of the lytic infection when p53 level was decreased interfered with viral genome synthesis (Figure 4C). These findings suggest that the degradation of p53 contributed to prevent apoptosis and was required for the efficient viral propagation in the lytic replication.

## Discussion

In this study, we revealed p53 to be degraded via an ubiquitin-proteasome pathway even under conditions of up-regulated ATM-dependent DNA damage signaling in the EBV lytic phase. Our data clearly indicate that the EBV BZLF1 IE protein plays a critical role in the degradation of p53 independent of MDM2. The BZLF1 protein interacts with Cul2 and Cul5 through the Cul2- and Cul5-box motifs, located within its N-terminus. BZLF1-Cul2/Cul5 complexes proved capable of reconstituting a multiprotein ECS complex with ubiquitin ligase activity.

The large body of evidence implicating Cul2- and Cul5-containing E3 ubiquitin ligases in regulation of diverse cellular processes [32] provides us with new insights into their significance as potential targets of viruses trying to manipulate the host cellular system. Several viral proteins have the capacity to assemble with Cullin-based ubiquitin ligase modules and act as E3 ligases. For instance, Vif encoded by HIV-1 interacts with Cul5 through a zinc-binding HCCH, a unique viral motif directing ubiquitin-mediated proteolysis of APOBEC3G, a host defense factor that causes hypermutation in newly synthesized viral DNA [33]. A major difference between the BZLF1 protein and these viral proteins is that BZLF1 protein associates not only with Cul2 but also with Cul5, indicating redundancy in the EBV machinery to downregulate p53. Although the molecular mechanisms are controversial, a variety of reports on the herpesvirus family have pointed to inactivation of p53 in lytic infection [34],[35],[36]. Consistently, overexpression of p53 in infected cells interferes with efficient expression of viral genes (data not shown). Given the importance of inhibiting p53-mediated transactivation to adapt the cellular environment for viral propagation, the apparent redundancy is not so surprising.

**Figure 5:**
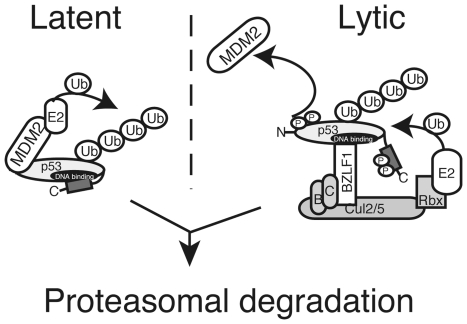
Model for p53 degradation in the EBV life cycle. With EBV latent infection, the level of p53 is regulated by MDM2 E3 ubiquitin ligase. Induction of lytic replication elicits the DNA damage response via activation of the ATM-dependent DNA damage signaling pathway. Under these conditions, p53 is hyperphosphorylated at S15 by ATM and S20, S366 and S378 by Chk2. C-terminal phosphorylation of p53 leads to allosteric conformational change through dissociation of the negative regulatory domain (gray) from the DNA binding domain (black), enhancing the binding of BZLF1 protein to p53. BZLF1 protein associated ECS ubiquitin ligase complexes then ubiquitinate p53, leading to proteasomal degradation during lytic infection.

The present study indicated the existence of distinct mechanisms of p53 quantitative regulation in the latent and lytic phases of EBV infection, as schematically illustrated in Figure 5. We observed that disruption of p53 binding to MDM2 by Nutlin-3 increased the level of p53 in latent phase but not during lytic infection [23]. Since induction of the EBV lytic program activates the ATM-Chk2 DNA damage-signaling pathway [21], p53 is phosphorylated at least at S15 and S20 but its level is nevertheless downregulated. Under these conditions, MDM2 hardly interacts with N-terminal phosphorylated p53 [4],[37], implying that EBV possesses another strategy to ubiquitinate phospho-p53 to block downstream signaling during lytic infection. On the basis of our findings with the BZLF1 protein and Cul2- and Cul5-containing ubiquitin ligase complexes, we propose a model for recognition and ubiquitination of p53 by the BZLF1 protein-associated E3 ligases (Figure 5). A requirement of these complexes for effective p53 degradation was supposed to achieve the efficient viral replication. In addition, the phosphorylation at S366 and S378 by virus-induced DNA damage response enhances the association with BZLF1 protein and ubiquitination of p53. To our knowledge, except for CARPs [38], neither RING nor HECT type E3 ligases [7] have previously been demonstrated to recognize and ubiquitinate phosphorylated p53 for degradation, including MDM2, COP1, Pirh2 or E6/E6AP. Thus, this finding is one of the most interesting aspects of our study.

Wen and colleagues revealed that BZLF1 is expressed as an immediate-early gene following primary EBV infection of B lymphocytes although early and late lytic gene expression is not observed [39]. They speculate that BZLF1-expressing cells are the only ones that survive and establish latency. In addition, it was reported that BZLF1 expression in early-passage lymphoblastoid cell lines may contribute to tumor formation in nudemice [40] and cellular gene expression [41]. Thus, it is noteworthy to mention the possibility that the degradation of p53 by BZLF1 protein-associated ECS ubiquitin ligases contributes to efficient establishment of latent infection at the early stages of primary EBV infection or tumor formation in vivo.

## Materials and Methods

### Cells and reagents

SaOS-2 cells, 293T cells, and 293/EBV cells were grown in DMEM supplemented with 10% fetal calf serum (FCS). 293/EBV cells were prepared by transfection with EBV-Bac DNA [42] into 293 cells by hygromycin selection (hygromycin B; 150 µg/ml). EBV-Bac was gifted by Wolfgang Hammerschmidt (Helmholtz Zentrum München-Haematologikum, Germany). EBV-positive marmoset B lymphocytes B95-8 cells and Tet-BZLF1/B95-8 cells were described previously [22]. For MG132 (Sigma) experiments, cells were treated with MG132 (20 µM) for 3–5 h, before harvesting. Anti-p53 (FL-393) and anti-Cul5 (H-300) rabbit polyclonal antibodies, anti-Rbx2 (N-15) goat polyclonal antibodies and normal mouse IgG2a were purchased from Santa Cruz Biotechnology. Anti-Phosho-p53 (Ser15), anti-Phosho-p53 (Ser20), anti-Phopho-Chk2 (Thr68) and anti-Cleaved PARP (Asp214) rabbit polyclonal antibodies were obtained from Cell Signaling Technology. Mouse anti-Elongin C (BD Transduction Laboratory), mouse anti-Elongin B (BioLegend), mouse anti-GAPDH (Ambion), monoclonal mouse anti-p53 (Ab-6) (Merck), mouse anti-MDM2 (Ab-3) (Merck) and mouse anti-FLAG M2 (Sigma) antibodies were also used. Anti-Cul2 rabbit polyclonal antibody and horseradish-peroxidase-conjugated secondary antibodies were purchased from Zymed Laboratories. Affinity-purified anti-BZLF1 and anti-Rbx1 antibodies were prepared as described previously [43]. Anti-phosho-p53 (Ser366) and (Ser378) antibodies were a generous gift from Sheau-Yann Shieh (Institute of Biomedical Sciences, Taiwan). These phospho-specific antibodies are used for immunoprecipitation, and are not suitable for immunoblot analysis.

### Plasmids and transfection

Mammalian expression vectors for human wild-type p53 (pcNXRS) and the BZLF1 protein expression vector (pcDNA-BZLF1) were kindly provided by Takashi Takahashi (Nagoya University, Japan) and Kiyotaka Kuzushima (Aichi Cancer Center Research Institute, Japan), respectively. Full-length cDNAs of BZLF1, Cul2, Cul5, and ubiquitin were obtained by reverse transcriptase-PCR (RT-PCR) and subcloned into the pcDNA4/TO/myc-His vector (Invitrogen). For expression of the epitope tagged protein, a FLAG-TEV-HA (FTH) or HA tag cassette was inserted into the plasmids encoding the respective cDNAs. For the expression of FLAG-tagged p53, constructs expressing p53 full-length wild-type (wtp53) and deletion mutants (p53/1–200, p53/100–292, p53/169–393, p53/309–368, p53/1–322̂323–393) in p3×FLAG-CMV-14 (Sigma) were prepared by PCR. BZLF1 mutants (M1, L44A P45A; M2, L52A P53A E54A P55A; M3, L44A P45A L52A P53A E54A P55A in pcDNA4A) and p53-mutant (L323A Y327A L330A; and S366A&S378A in p3×FLAG-CMV-14 and S15E; S15&S37E; S366E; S378E and S366E&S378E in pET28b (Novagen)) expression plasmids were generated by site-directed mutagenesis. A BZLF1 deletion mutant (BZLF1 d200-227), lacking part of the Zip domain, was generated by overlapping PCR. The inserted DNA sequence of each vector was confirmed by direct DNA sequencing.

Cells were seeded, cultured to semi-confluence and transfected with expression plasmids using lipofection reagent (Lipofectamine™ and Plus reagent; Invitrogen) according to manufacturer's instructions.

### RNAi

Knockdown of Cul2 or Cul5 was achieved by electroporation with shRNA plasmids as described previously [14]. Two days after transfection, cells were harvested and subjected to immunoblotting.

### Immunoprecipitation and Western blotting

Cells were lysed in lysis buffer (50 mM Tris-HCl pH 7.6, 120 mM NaCl, 0.1% NP40, 1 mM EDTA, 100 mM sodium fluoride, 2 mM sodium vanadate) containing a protease inhibitor cocktail (Sigma), and then sonicated. The debris was removed by centrifugation and the supernatants were applied for immunoprecipitation with specific antibodies. Complexes of antibody and antigen were collected by centrifugation and washed three times with NET-gel buffer (50 mM Tris-HCl pH 7.6, 150 mM NaCl, 0.1% NP40, 1 mM EDTA). The immunoprecipitates were then subjected to SDS-PAGE followed by immunoblot analyses. Preparation of the lysate for immunoblotting, Western blotting and detection of signals were performed as described previously [44]. Immunoreactivity was detected by Western Lightning (Perkin-Elmer) and images were processed with LumiVision PRO 400EX (Aisin/Taitec Inc.). Signal intensity was quantified with a LumiVision Analyzer 400. The system used in this study mounts a cooled CCD camera that has a 16 bit = 65,535 grayscale wide dynamic range. It enhances the accuracy of the quantitative analysis up to 100 times compared with ordinary quantitative analysis scanning of an X-ray film into the personal computer after exposing the signal to the film. Protein levels were quantified by the densitometry in triplicate experiments, and the results were expressed as ratios between the specific band under examination and appropriate internal control.

### Purification of proteins and in vitro ubiquitination assays

For preparing reconstituted RHF-ECS complexes, lysates from Sf21 cells co-infected with baculoviruses encoding RHF-BZLF1, HA-Cul2/5, myc-Rbx1/2, Elongin B and Elongin C, were applied to Ni-nitrilotriacetic acid agarose (QIAGEN) as described previously [16],[25]. HA-tagged p53 was expressed in Sf21 cells infected with a recombinant baculovirus [45], kindly provided by Carol Prives (Columbia University). HA-p53 protein was purified using a monoclonal anti-HA agarose conjugate (Sigma) and elution was performed using an HA peptide. To prepare His-tagged p53, His-p53 and its phospho-mimetic mutants were expressed in bacteria, and the expressed proteins were purified using Ni-nitrilotriacetic acid agarose. In vitro ubiquitination assays were performed as described previously [16] with some modifications. Reaction mixtures were incubated for 1 h at 26°C, separated by SDS-PAGE, and analyzed by IB with anti-p53 (Ab-6) antibody.

### Purification of EBV BZLF1 protein

Hi-Five cells were infected with recombinant baculovirus AcBZLF1, harvested 72 h post-infection, and then suspended in hypotonic buffer (HB; 40 mM Tris-HCl pH 7.6, 1 mM EDTA, 1 mM EGTA, 1 mM DTT, 1 mM PMSF, 0.2% Triton X-100, 10 µg/ml leupeptin, 10 µg/ml pepstatin), followed by homogenization using a Dounce homogenizer. The resultant nuclei were freeze and thawed, resuspended in 0.1 M NaCl-HB, and precipitated again. After washing with 0.2 M NaCl-HB twice, extraction of the BZLF1 protein was performed with 0.6 M NaCl-HB. The purity of the recombinant BZLF1 protein is more than 90%.

### Quantification of viral DNA synthesis during lytic replication

Tet-BZLF1/B95-8 cells (1×10^6^ cells) were treated with doxycycline and then transfected with p53 expression plasmid or empty plasmid as a control at the indicated times. Total DNAs were purified from the cells at 48 h post-induction. Dot-blot hybridization was performed using DIG-labeling system (Roche) and viral genome replication was quantified as described previously [22].

### Titration of virus yields from 293/EBV cells

For titration of virus yields, 293/EBV cells were transfected with BZLF1 expression plasmid using a microporator (Digital Bio) to induce lytic replication. Cells and the culture supernatant were collected, freeze-thawed, and centrifuged. The supernatant from the centrifugation was filtered and used as a virus stock. EBV-negative Akata (-) cells [46] (kindly provided by Kenzo Takada, Hokkaido University) were infected with the virus and EGFP positive cells were counted by FACS.

### Data analysis and statistics

Data are presented as mean±S.E.. Statistical analysis has been carried out using Student's *t*-test Values were considered significantly different when *p*<0.05.

### Accession numbers

The Entrez Gene accession numbers for genes and gene products discussed in this study are as follows: p53 (7157), ubiquitin (7314), Cul2 (8453), Cul5 (8065), and BZLF1 protein (3783744).

## Supporting Information

Figure S1 Inhibition of the p53 reduction shows negative effect on the EBV replication and viral gene expression. (A) BZLF1 protein interacts with either Cul2 or Cul5. Lysates of 293T cells transiently expressing the indicated proteins were subjected to immunoprecipitation followed by immunoblotting with the indicated antibodies. (B) BZLF1 protein interacts with p53 and Elongin C, a component of ECS complex in lytic replication-induced cells. The lytic infection was induced by transfection with BZLF1 protein expression plasmid into 293/EBV cells. Cells were treated with MG132 for 4 h before harvesting and then harvested 48 h post-transfection. IP and IB assays were performed using the indicated antibodies. (C) BZLF1 protein interacts with both Cul2 and Cul5 in lytic replication-induced cells. Tet-BZLF1/B95-8 cells were cultured with Dox for 24 h and then treated with MG132 for 3 h before harvesting. IP and IB assays were performed using the indicated antibodies.
(0.20 MB PDF)

Figure S2 A series of in vitro ubiquitination assays. (A) The BZLF1 protein is essential for ubiquitination of p53 by ECS complexes. Individual protein complexes were purified from Sf21 cells co-infected with recombinant baculoviruses encoding these components. These complexes were assayed for their ability to mediate the ubiquitination of p53 in the presence of ATP, Uba1 (E1), UbcH5A (E2) and GST-Ub. Reaction mixtures were incubated for 1 h at 26°C, boiled in SDS sample buffer, and then subjected to IB analysis with anti-p53 antibody. Intensity of the polyubiquitin chains is expressed as a ratio between polyubiquitinated p53 protein and p53 protein nput. (B and C) Both BZLF1-Cul2 and BZLF1-Cul5 complexes allow ubiquitination of p53 in vitro. Reaction mixtures were incubated for the indicated times. (D and E) Both BZLF1-Cul2 and BZLF1-Cul5 complexes allow ubiquitination of p53 in vitro. A drop out assay was carried out to determine the specificity of in vitro p53 ubiquitination. (F) The BZLF1 protein acts as an adaptor protein to recognize the substrate for p53 ubiquitination. Wild type BZLF1 (WT) or d200-227 mutant protein was expressed with components of the EC_2_S complex in Sf21 cells, and cell lysates were subjected to Ni-NTA affinity purification. The purified BZLF1 complexes were applied to the reaction and then IB analysis with anti-p53 antibody (right panels). Each recombinant BZLF1-EC_2_S complex purified from Sf21 insect cell lysates was analyzed by IB with the indicated antibodies (left panels). (G) The phospho-mimic p53 mutant is also more ubiquitinated than wild-type p53 by BZLF1-EC_2_S complex in vitro. Recombinant His-p53 protein (WT or S366E&S378E) was incubated in a reaction mixture containing purified BZLF1-EC_2_S complex.
(0.62 MB PDF)
