# Supplementary figures and images for: Degradation of Phosphorylated p53 by Viral Protein-ECS E3 Ligase Complex
Source: PLoS Pathog. 2009 Jul 31;5(7):e1000530. doi: 10.1371/journal.ppat.1000530 (PMC2712087; doi:10.1371/journal.ppat.1000530)

**A**Input(2%) IP: $\alpha$ -FLAG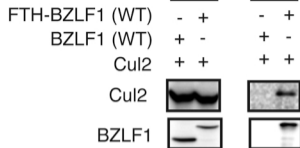Input(2%) IP: $\alpha$ -FLAG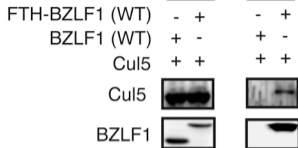**B**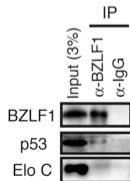**C**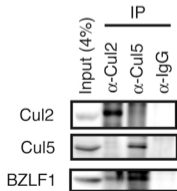

Supplement: Figure S1 — Inhibition of the p53 reduction shows negative effect on the EBV replication and viral gene expression. (A) BZLF1 protein interacts with either Cul2 or Cul5. Lysates of 293T cells transiently expressing the indicated proteins were subjected to immunoprecipitation followed by immunoblotting with the indicated antibodies. (B) BZLF1 protein interacts with p53 and Elongin C, a component of ECS complex in lytic replication-induced cells. The lytic infection was induced by transfection with BZLF1 protein expression plasmid into 293/EBV cells. Cells were treated with MG132 for 4 h before harvesting and then harvested 48 h post-transfection. IP and IB assays were performed using the indicated antibodies. (C) BZLF1 protein interacts with both Cul2 and Cul5 in lytic replication-induced cells. Tet-BZLF1/B95-8 cells were cultured with Dox for 24 h and then treated with MG132 for 3 h before harvesting. IP and IB assays were performed using the indicated antibodies. (0.20 MB PDF) [file ppat.1000530.s001.pdf]

**A**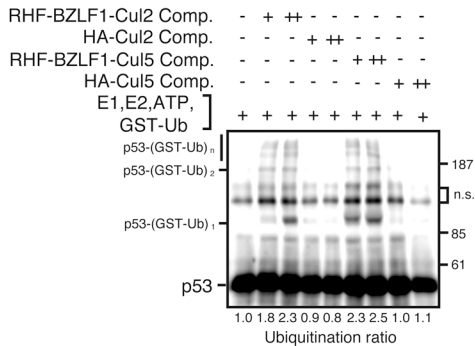**B**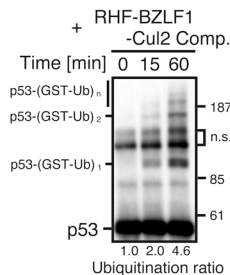**C**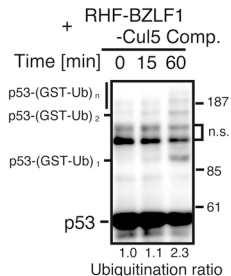**D**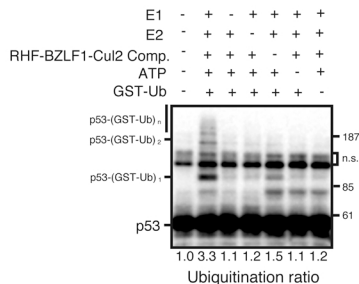**E**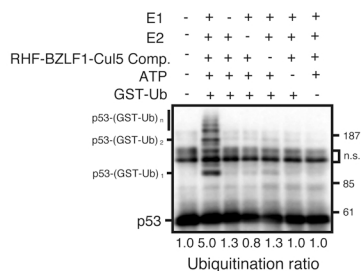**F**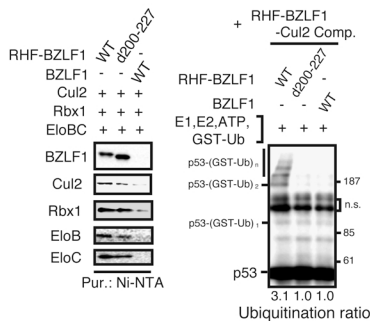**G**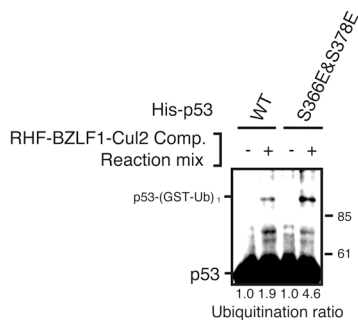

Supplement: Figure S2 — A series of in vitro ubiquitination assays. (A) The BZLF1 protein is essential for ubiquitination of p53 by ECS complexes. Individual protein complexes were purified from Sf21 cells co-infected with recombinant baculoviruses encoding these components. These complexes were assayed for their ability to mediate the ubiquitination of p53 in the presence of ATP, Uba1 (E1), UbcH5A (E2) and GST-Ub. Reaction mixtures were incubated for 1 h at 26°C, boiled in SDS sample buffer, and then subjected to IB analysis with anti-p53 antibody. Intensity of the polyubiquitin chains is expressed as a ratio between polyubiquitinated p53 protein and p53 protein nput. (B and C) Both BZLF1-Cul2 and BZLF1-Cul5 complexes allow ubiquitination of p53 in vitro. Reaction mixtures were incubated for the indicated times. (D and E) Both BZLF1-Cul2 and BZLF1-Cul5 complexes allow ubiquitination of p53 in vitro. A drop out assay was carried out to determine the specificity of in vitro p53 ubiquitination. (F) The BZLF1 protein acts as an adaptor protein to recognize the substrate for p53 ubiquitination. Wild type BZLF1 (WT) or d200-227 mutant protein was expressed with components of the EC2S complex in Sf21 cells, and cell lysates were subjected to Ni-NTA affinity purification. The purified BZLF1 complexes were applied to the reaction and then IB analysis with anti-p53 antibody (right panels). Each recombinant BZLF1-EC2S complex purified from Sf21 insect cell lysates was analyzed by IB with the indicated antibodies (left panels). (G) The phospho-mimic p53 mutant is also more ubiquitinated than wild-type p53 by BZLF1-EC2S complex in vitro. Recombinant His-p53 protein (WT or S366E&S378E) was incubated in a reaction mixture containing purified BZLF1-EC2S complex. (0.62 MB PDF) [file ppat.1000530.s002.pdf]
